# Exposure of *Helicobacter pylori* to clarithromycin *in vitro* resulting in the development of resistance and triggers metabolic reprogramming associated with virulence and pathogenicity

**DOI:** 10.1371/journal.pone.0298434

**Published:** 2024-03-06

**Authors:** Naim Asyraf Rosli, Anis Rageh Al-Maleki, Mun Fai Loke, Sun Tee Tay, Mohd Salleh Rofiee, Lay Kek Teh, Mohd Zaki Salleh, Jamuna Vadivelu

**Affiliations:** 1 Faculty of Medicine, Department of Medical Microbiology, Universiti Malaya, Kuala Lumpur, Malaysia; 2 Faculty of Medicine and Health Sciences, Department of Medical Microbiology, Sana’a University, Sana’a, Yemen; 3 Camtech Biomedical Pte Ltd, Singapore, Singapore; 4 Integrative Pharmacogenomics Institute (iPROMISE), Universiti Teknologi MARA, Selangor, Malaysia; 5 Faculty of Medicine, Medical Education Research and Development Unit (MERDU), Universiti Malaya, Kuala Lumpur, Malaysia; Federal University Dutse, NIGERIA

## Abstract

In *H*. *pylori* infection, antibiotic-resistance is one of the most common causes of treatment failure. Bacterial metabolic activities, such as energy production, bacterial growth, cell wall construction, and cell-cell communication, all play important roles in antimicrobial resistance mechanisms. Identification of microbial metabolites may result in the discovery of novel antimicrobial therapeutic targets and treatments. The purpose of this work is to assess *H*. *pylori* metabolomic reprogramming in order to reveal the underlying mechanisms associated with the development of clarithromycin resistance. Previously, four *H*. *pylori* isolates were induced to become resistant to clarithromycin *in vitro* by incrementally increasing the concentrations of clarithromycin. Bacterial metabolites were extracted using the Bligh and Dyer technique and analyzed using metabolomic fingerprinting based on Liquid Chromatography Quadrupole Time-of-Flight Mass Spectrometry (LC-Q-ToF-MS). The data was processed and analyzed using the MassHunter Qualitative Analysis and Mass Profiler Professional software. In parental sensitivity (S), breakpoint isolates (B), and induced resistance isolates (R) *H*. *pylori* isolates, 982 metabolites were found. Furthermore, based on accurate mass, isotope ratios, abundances, and spacing, 292 metabolites matched the metabolites in the Agilent METLIN precise Mass-Personal Metabolite Database and Library (AM-PCDL). Several metabolites associated with bacterial virulence, pathogenicity, survival, and proliferation (L-leucine, Pyridoxone [Vitamine B6], D-Mannitol, Sphingolipids, Indoleacrylic acid, Dulcitol, and D-Proline) were found to be elevated in generated resistant *H*. *pylori* isolates when compared to parental sensitive isolates. The elevated metabolites could be part of antibiotics resistance mechanisms. Understanding the fundamental metabolome changes in the course of progressing from clarithromycin-sensitive to breakpoint to resistant in *H*. *pylori* clinical isolates may be a promising strategy for discovering novel alternatives therapeutic targets.

## Introduction

Metabolomic reprogramming refers to the ability of cells to alter their metabolome and phenotypic characteristics in response to environmental changes [1,2]. Metabolites are intermediate molecules and by-products of metabolic catalysis [3]. A key component of cellular regulatory system is metabolites produced by bacteria that interacts with the surrounding host microenvironment [4,5]. Several studies have shown that in *Staphylococcus aureus* and *Vibrio alginolyticus*, phenotypic changes, including antimicrobial resistance, can be associated with metabolomic reprogramming [6,7]. Antibiotic resistance has been demonstrated to correlate with alterations in bacterial metabolic pathway, such as folate biosynthesis pathway which is associated with changing bacterial susceptibility to antibiotics (phenotypic resistance) [8]. Moreover, the acquisition of resistance is shown in in *Escherichia coli* and *S*. *aureus* to impact bacterial physiology due to increased metabolomic burden and fitness cost [9]. These reports are supported by the other findings that acquiring phenotypic resistance requires the concerted activity of many components that are important in microbial physiology, including electron transport chain and metabolism of amino acids, fatty acids, or nucleotides [7,9–11].

*Helicobacter pylori* is a Gram-negative microaerophilic bacterium that causes gastric diseases, such as gastritis and peptic ulcers, and is associated with stomach cancer [12,13]. The ability of *H*. *pylori* to adapt and thrive in the harsh acidic environment of the stomach is one of the most remarkable elements of its biology [14]. *H*. *pylori* accomplishes this via producing a variety of metabolites, such as cholesterol-derived metabolites [15]. These compounds are critical in the bacterium’s capacity to colonize and maintain infection, avoid detection by the host immune system, and interact with the host stomach epithelium [15,16]. In addition, the metabolic status of bacterial cells exposed to antibiotics may directly or indirectly result in antimicrobial resistance (AMR) [17]. Reduced metabolic activity can lead to AMR by limiting antibiotic absorption [18,19]. In contrast, high metabolic activity is essential to sustain energy-demanding AMR processes, such cell-wall changes and efflux pump overexpression [9,20–22].

Clarithromycin-based triple treatment remains the therapy of choice for treating *H*. *pylori* infection [23]. Clarithromycin, a macrolide antibiotic, is still an effective antibiotic used in many *H*. *pylori* treatment regimens [24]. Clarithromycin interacts with 50S ribosomal subunit of *H*. *pylori*, leading to the suppressing of protein synthesis and resulting in a bacteriostatic effect [25]. However, metabolomic reprograming and metabolites associated with the development of clarithromycin-resistance in *H*. *pylori* is not well studied yet. Therefore, in this study, the *H*. *pylori* metabolomic reprogramming was assessed to elucidate the underlying mechanisms associated with *in vitro* induced development of clarithromycin-resistance by comparing the metabolomic profiles in the course of progressing from clarithromycin-sensitive to breakpoint to resistance *H*. *pylori* clinical isolates.

## Materials and methods

### *H*. *pylori* parental and induced isogenic isolates

Four *H*. *pylori* isolates have been retrieved from the collection of the *Helicobacter* Research Laboratory (Marshall Centre), HIR at the Universiti Malaya. Ethics approval was not required since no human participants and/or medical data were involved in the investigation. Four clarithromycin-sensitive *H*. *pylori* isolates were induced by exposing them to sequentially increasing concentrations of clarithromycin ranging from 0.0156 g/mL to 32 g/mL incorporated in CA plates in our previous study [26]. The parental uninduced clinical isolates were labeled as Sensitive (S), the induced isolates collected one passage immediate to becoming resistant were labeled as Breakpoint (B), and the induced resistance isogenic isolates were labeled Resistant (R). Isolates that grew on plate containing >1 μg/mL clarithromycin were considered as resistant. The isolates were grouped based on their clarithromycin susceptibility into S isolates (UM171-S, UM626A1-S, and UM678A-S with MIC of 0.064 μg/mL and UM650B-S with MIC of <0.016 to 0.064 μg/mL, B isogenic isolates (UM171-B, UM UM626A1-B, UM650B-B, and UM678A-B with MIC of 0.125 μg/mL), and R isogenic isolates (UM171-R, UM626A1-R, UM650B-R, and UM678A-R with MIC of >64 μg/mL). Comparing B isolates to those of R and S isolates allows us to provide insight on metabolomic reprogramming which has previously received little attention. The MIC fold changes of B and R compared to the parental S isolates are shown in S1 Fig.

### Microbiological conditions for culturing

All *H*. *pylori* isolates were grown on chocolate agar (CA) (Oxoid, UK) for three days in a humidified 10% CO2 incubator at 37 °C (Thermo Fisher Scientific, USA) [27]. For broth culture, Brain heart infusion (BHI) broth (Oxoid, UK) supplemented with 0.4% yeast extract (Oxoid, UK) and 1% β-Cyclodextrin (Sigma, USA) was used.

### Preparation of metabolite samples

The *H*. *pylori* colonies from a non-selective CA plate were harvested and inoculated into BHI broth before being incubated at 37 °C in a 10% CO_2_ incubator for another three days to reach log growth phase. The bacterial cultures were then adjusted with BHI broth to an OD600 nm of 1.0. Subsequently, 500 μL of the adjusted bacterial suspensions were pelleted at 10,000 rpm for one minute and washed three times with fresh BHI broth. The metabolites were extracted from the bacterial pellet using the Bligh and Dyer extraction procedure [28]. In brief, 300 μL of 2:1 methanol-chloroform (v/v) was added to each bacterial pellet and vortexed for a minute before incubating at room temperature for an hour. Following that, 200 μL of 1:1 chloroform-water (v/v) obtained from the Milli-Q® Benchtop Lab Water Purification Systems (Sigma-Aldrich, USA) were added to the mixture. The extract was then incubated at room temperature for 10 minutes and centrifuged at 10,000 rpm for ten minutes at 4 °C. the upper aqueous and lower organic phases were harvested and dried at 4 °C using the Labconco Refrigerated Centrivap concentrator (Kansas City, MO, USA).

### LC-Q-TOF analysis of metabolites

The analysis of metabolites was performed by the method described previously with modification [28,29]. Briefly, the dried samples were redissolved in 50 μl of acetonitrile and water. The samples were mass spectrometrically analyzed on a 1200 Infinity Quaternary Liquid Chromatography system outfitted with a 6520 Quadrupole Time-of-Flight mass spectrometer attached to a Dual Agilent Jet Stream Electrospray Ionization (Dual AJS ESI) ionization source (Agilent Technologies, CA, USA). The samples were separated using an Agilent Zorbax Eclipse plus C18 Rapid Resolution High Throughput separation column (2.1 100 mm 1.8 m), kept at 40 °C to 45 °C, and analyzed in positive mode. Water with 1% formic acid (A) and acetonitrile with 0.1% formic acid (B) were utilized as mobile phases. A linear gradient from 95% to 5% was set to run for 36 minutes, with 5% A held for 5 minutes and flushed for 7 minutes at 0.5 mL/min. In the event of the run, the injection volume was set to 2 μL, and three injections were performed in complete loop injection mode for each sample. For instrument control and data collection, Agilent MassHunter Workstation Data Acquisition software (Agilent Technologies, CA, USA) was utilized. The nebulizer gas pressure (nitrogen) was set to 160 kPa, the ion source temperature was set to 200°C, the dry gas flow was set to 7 mL/minute at source temperature, and the spectral rate was set to 3 Hz for MS1 and 10 Hz for MS2. Voltage 3.0 kV, gas temperature 300 °C, drying gas 8 L/min, nebulizer 35 psig, VCap 3500 V, fragmentor 175 V, and skimmer 65 V were the electrospray ionization (ESI) conditions. To ensure high-quality data in metabolite profiling, sample blanks were run after each sample to clean the column and prevent previous samples from being carried over.

### Metabolomics data processing and statistical analysis

The metabolome analysis of S, B, and R groups of *H*. *pylori* isolates were performed as described previously [28]. For the qualification and quantification of metabolites, the MassHunter Qualitative Analysis (MQA) software (Qual; version R.06.00) (Agilent Technologies, CA, USA) removes convolution of the raw data into individual chemical peaks for Molecular Feature Extraction. Moreover, we identified features with a minimum absolute abundance of 1000 counts within a predetermined mass accuracy (+/-5 ppm). The produced data were then imported into the Agilent Mass Profiler Professional (MPP) software (version B.12.61) for binning, alignment, and consensus generation for each feature. The data was imported back into MPP for annotation, statistical analysis, and comparison of S, B, and R groups’ metabolites. The abundance for each feature was normalized to the median abundance across all samples using MPP’s baselining option. The inherent variation between the S, B, and R groups was visualized using unsupervised principal component analysis (PCA) with median centering and scaling. One-way ANOVA was used to identify the metabolites that vary significantly between any two groups at a fold change (FC) cutoff value of 2 at *p*-value <0.05 with the Benjamini and Hochberg False Discovery Rate (FDR) adjusted to 1% for multiple testing adjustments. Furthermore, *t-*test was also applied to calculate the significance and FC of the metabolites between two groups. The metabolites were identified using MPP’s IDBrowser function by comparing mined features based on accurate mass, isotope ratios, abundances, and spacing to metabolites in the Agilent METLIN Accurate Mass-Personal Metabolite Database and Library (AM-PCDL) database (version 5.0). Subsequently, the identified metabolites were further analyzed for their correlation to the development of antibiotic resistance in *H*. *pylori* by comparing the metabolites expressed in B and R isolates with the parental S isolates.

## Results

### Global metabolome profile

**Fig 1:**
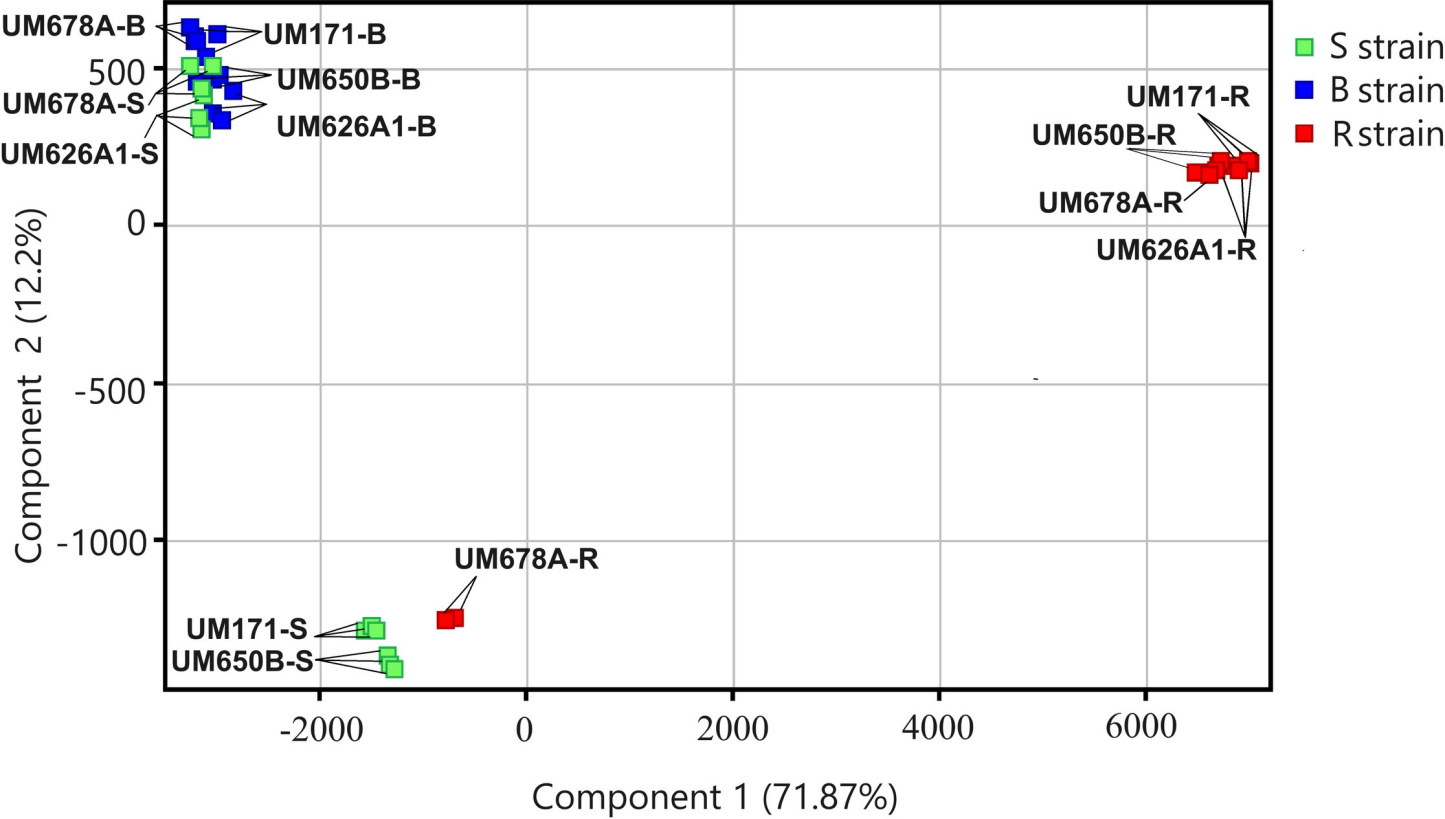
PCA in two dimensions of 12 *H*. *pylori* isolates created using MPP. 12 S isolates (4 isolates x 3 triplicate = 12); dark green color squares, 12 B isolates (4 isolates x 3 triplicate = 12); blue color squares, and 12 R isolates (4 isolates x 3 triplicate = 12) red color squares. *H*. *pylori* isolates formed distinct clusters based on metabolites acquired in positive ionization mode using components 1 (71.87%) and 2 (12.2%).

Unsupervised PCA was used for quality control of samples. PCA is most frequently used to compress, with the least amount of information loss, the information present in a large number of original variables into a smaller set of new composite dimensions [30]. The first two principal components of the PCA are represented by x axis (principal component 1, PC1) and y axis (principal component 2, PC2). PC1 reveals the maximum variation among data points, while PC2 reveals the second most variation. The values on each axis show the proportion of variance that each component accounts for. For global metabolomic profiles (i.e., all molecular characteristics without filtering using Euclidean distance matrix), the B isolates (blue) formed a closely clustered group on the PCA plot. UM626A1-S and UM678A-S (green) clustered closely with the B isolates while UM650B-S and UM171S formed a separate cluster, which is close to the B cluster along PC1. While UM171-R, UM626A1-R, and UM650B-R formed a distinctly separate cluster along PC1 away from the S and B cluster, UM678A-R clustered together with UM650B-S and UM171-S (Fig 1). Thus, each group has a distinct global metabolomic profile that could be examined further in the study.

A total of 982 metabolites were identified in this study. Notably, using one-way ANOVA, 593 metabolites were significantly (*p* <0.05) upregulated in R compared to B. However, 3 metabolites were not significantly changed in these two groups. Moreover, 585 metabolites were significantly (*p* <0.05) upregulated in R isolates against S isolates. However, 5 metabolites were not significantly changed in these two groups. Interestingly, 568 metabolites were not significantly changed in B and S groups. In contrast, only 249 metabolites were significantly (*p* <0.05) upregulated in B isolates against S isolates. Thus, there were more metabolites that were significantly upregulated as the *H*. *pylori* isolates progressed from sensitive to breakpoint and eventually to resistant.

**Fig 2:**
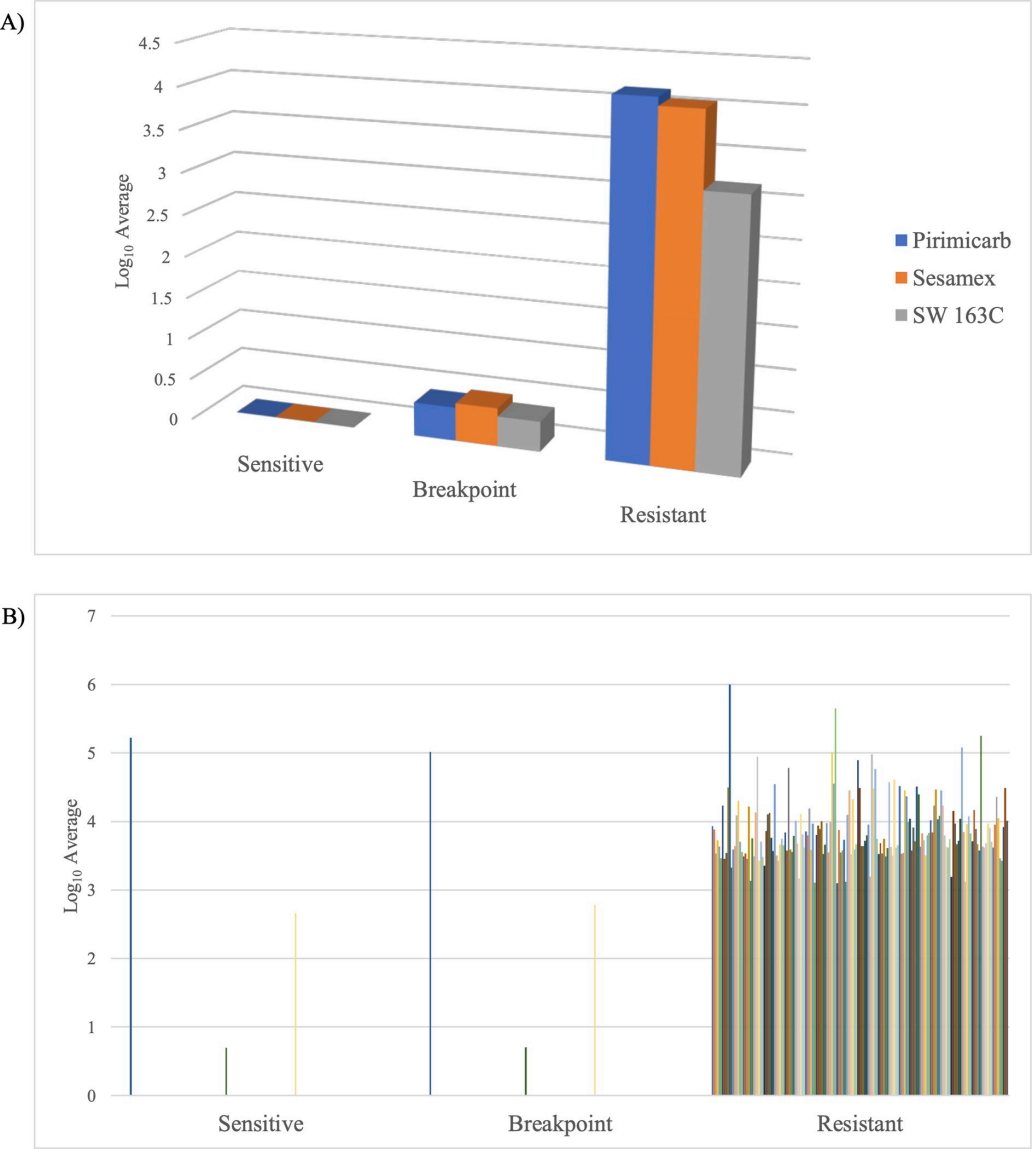
The regulation of metabolites across S, B, and R isolates. A) The metabolites were significantly upregulated (p < 0.05, FC > 2) in R isolates against B and S isolates, and significantly upregulated (p < 0.05, FC > 2) in B isolates against S isolates. B) 171 metabolites (S1 Table) were significantly upregulated (p < 0.05, FC > 2) in R isolates against B and S isolates, however there were no significant difference between those metabolites in B and S isolates.

**Fig 3:**
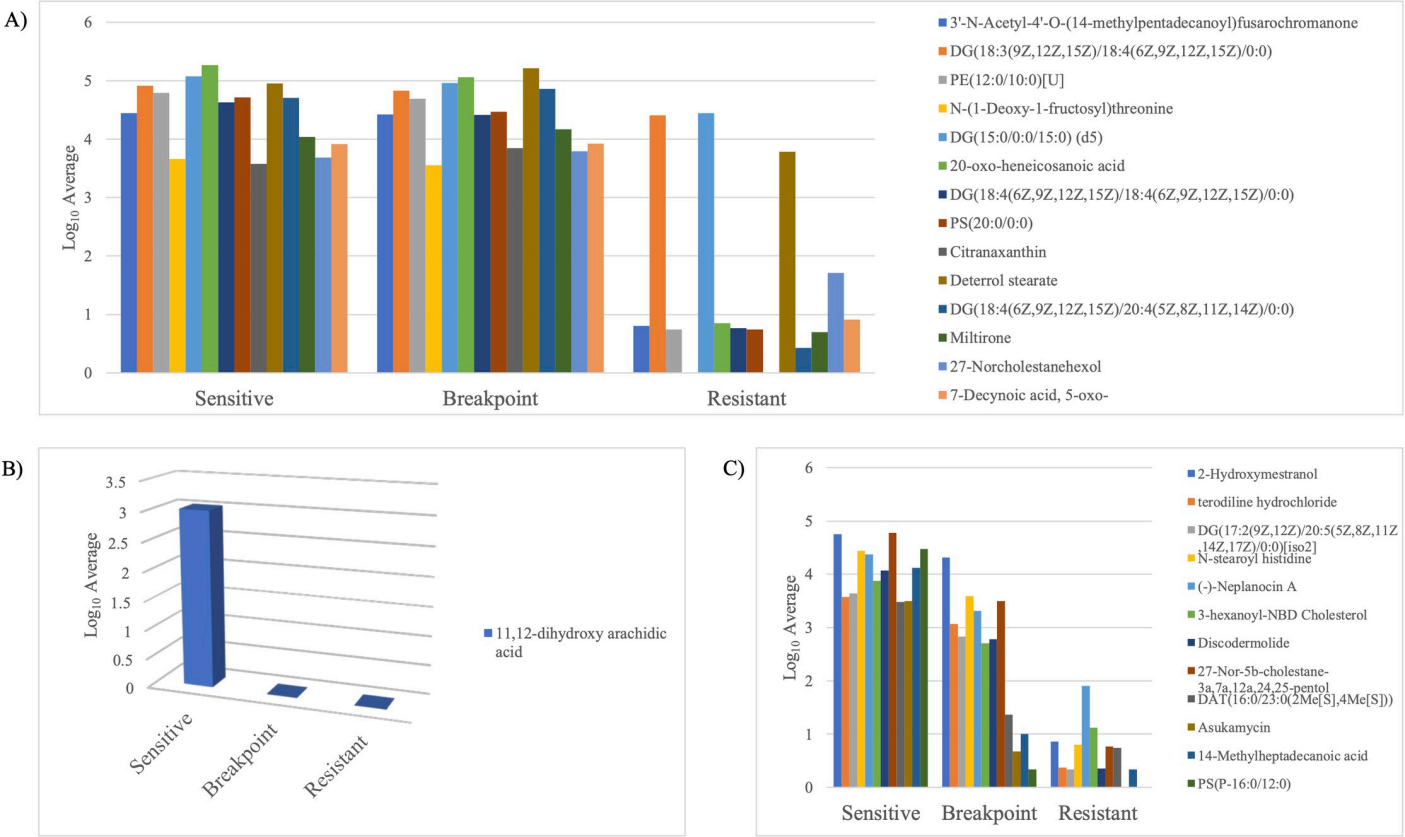
The regulation of metabolites across S, B, and R isolates. A) The metabolites were significantly downregulated (p < 0.05, FC < -2) in R isolates against B and S isolates, however there were no significant difference between those metabolites in B and S isolates. B) The metabolites were significantly downregulated (p < 0.05, FC < -2) in B and R isolates against S, however, there were no significant difference between those metabolites in B and R isolates. C) The metabolites were significantly downregulated (p < 0.05, FC < -2) in R isolates against B and S isolates, and significantly downregulated (p < 0.05, FC < -2) in B isolates against S isolates.

**Fig 4:**
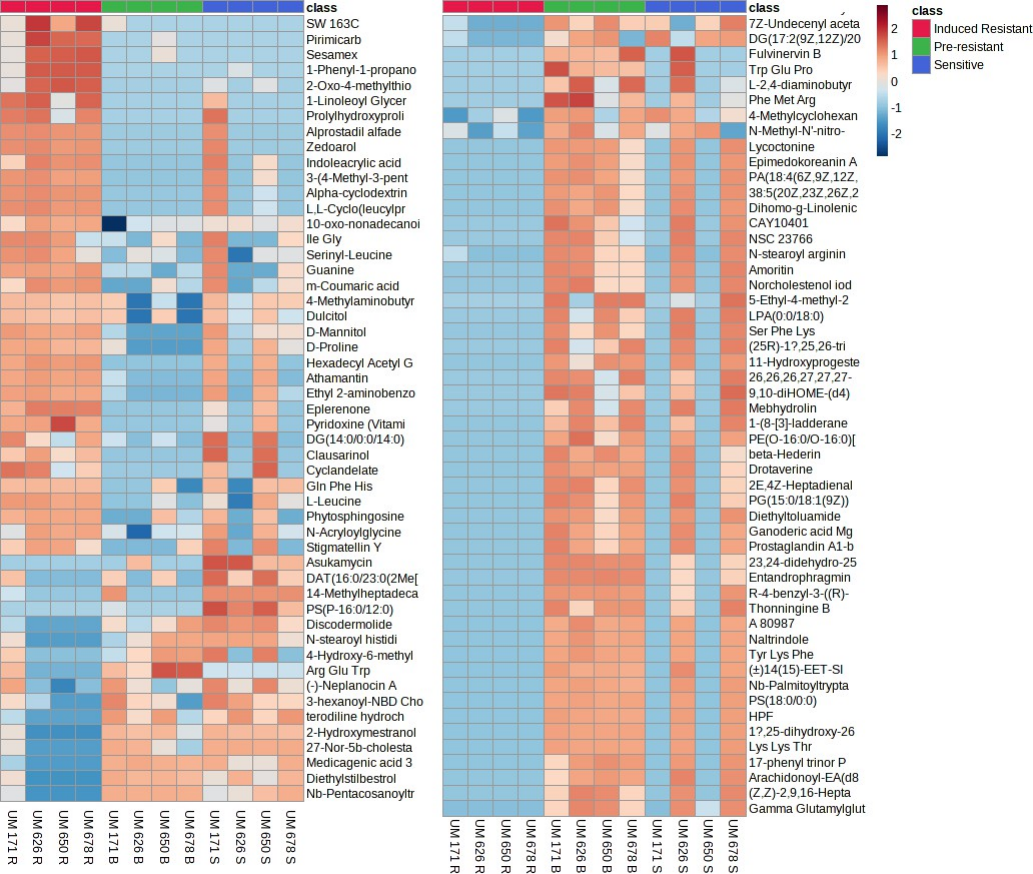
Heat map and clustering presenting the significant metabolome profiles of the 12 individual (S, B, and R) *H*. *pylori* isolates based on identified metabolites. The heat maps and clustering based on Euclidean distance were generated using MetaboAnalyst version 5. The metabolites regulation was significant with *p* <0.05 and fold change of 2 as a cutoff.

Furthermore, the metabolites were matched with metabolites in the AM-PCDL database based on accurate mass, isotope ratios, abundances and spacing to identify the metabolites. Among the 982 metabolites identified, 292 metabolites matched metabolites in the database (S1 Table). Interestingly, among the 292 metabolites, 3 metabolites were significantly upregulated (p < 0.05, FC > 2) in R against B and S isolates (Fig 2A), and significantly upregulated (p < 0.05, FC > 2) in B isolates against S isolates. Moreover, there were 171 metabolites significantly upregulated (p < 0.05, FC > 2) in R isolates against B and S, however there were no significant difference between B isolates and S isolates (Fig 2B). Nonetheless, 14 metabolites were significantly downregulated (p < 0.05, FC < -2) in R isolates against B and S isolates, however there were no significant difference between those metabolites in B and S isolates (Fig 3A). Notably, 1 metabolite (11,12-dihydroxy arachidic acid) was significantly downregulated (p < 0.05, FC < -2) in B and R isolates against S, however, there were no significant difference between those metabolites in B and R isolates (Fig 3B). Additionally, it is interesting to note that 12 metabolites were significantly downregulated (p < 0.05, FC < -2) in R isolates against B and S isolates, and significantly downregulated (p < 0.05, FC < -2) in B isolates against S isolates (Fig 3C). Fig 4 depicts the metabolites regulation among the 12 individual isolates with a significant of p <0.05 and fold change of 2 as a cutoff.

### Metabolic pathway profiles

**Fig 5:**
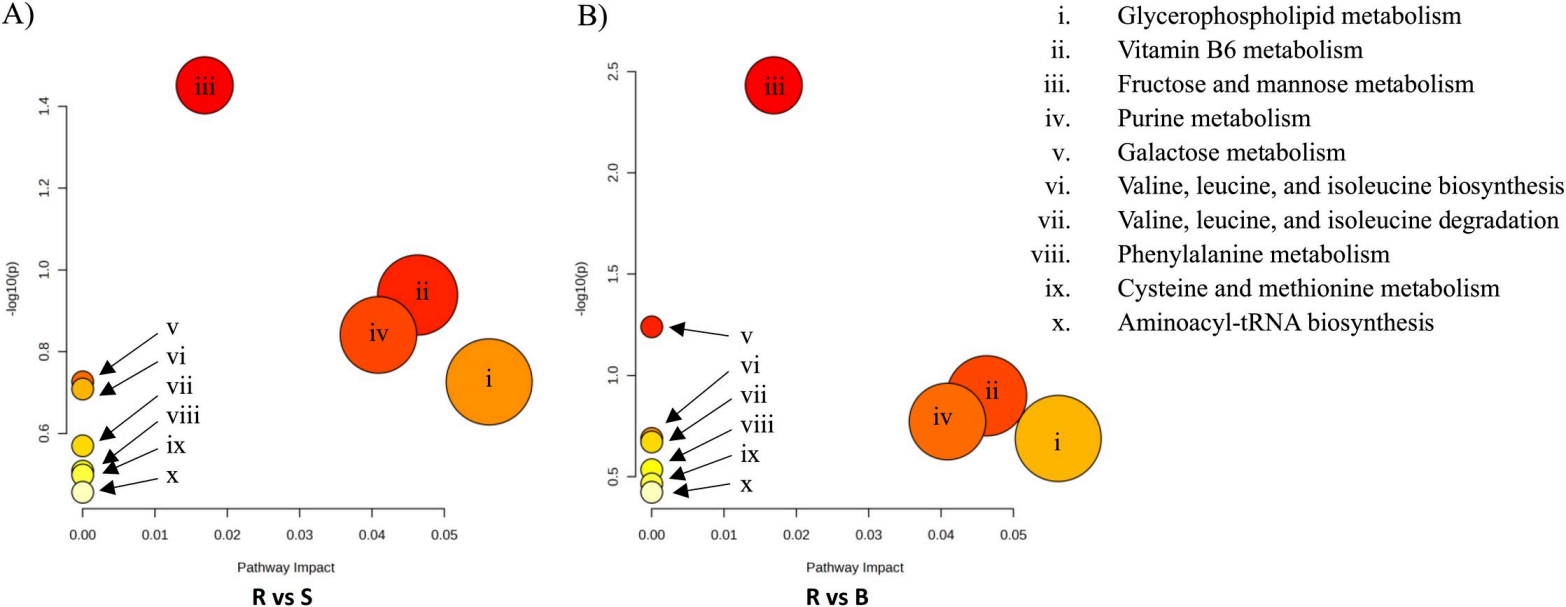
Overview of the pathway analysis which may be associated with the development of antibiotic resistance in *H*. *pylori* using MetaboAnalyst 5.0. The figure displays pathways that correspond to annotated metabolites and are organized according to the p-value (y-axis), which represents pathway enrichment analysis, and the pathway impact values (x-axis), which represents pathway topology analysis. The metabolic pathways are shown as bubbles based on their scores from enrichment as seen in the y axis, and topology (pathway impact) values as seen in x axis. The p-value determines the node color of each pathway (red = lowest p-value and highest statistical significance) and indicates that metabolite changes in the relevant pathway are more significant. The size of the bubble correlates to the pathway impact score (biggest indicating the highest impact) and is related to the centrality of the metabolites involved. Specify pathway analysis parameters include Relative-betweenness Centrality for topology analysis and Hypergeometric Test for enrichment method. The KEGG pathway has been selected as the library pathway. A) Pathway analysis of metabolites expressed in R isolates against S isolates, B) Pathway analysis of metabolites expressed in R isolates against B isolates.

The identified metabolites were mapped to metabolic pathways in the Kyoto Encyclopedia of Genes and Genomes (KEGG) database. Notably, metabolites up-regulated in R isolates against S isolates were mapped to 53 metabolic pathways, while only 20 metabolic pathways were mapped by the down-regulated metabolites (S2 Fig). Similarly, metabolites up-regulated in R isolates against B isolates were mapped to 58 metabolic pathways, whereas down-regulated metabolites were mapped to only 20 metabolic pathways (S4 Fig). Additionally, up-regulated metabolites in B isolates against S isolates were mapped to 20 metabolic pathways, whereas 36 metabolic pathways were mapped by the down-regulated metabolites (S3 Fig). The metabolic pathway was further analyzed with MetaboAnalyst 5.0. The up-regulated metabolites among R isolates against B and S isolates were mapped to 10 metabolic pathways (Table 1 and Fig 5). The results of metabolic pathway analysis demonstrate that the up-regulated metabolites that associated with clarithromycin resistance among R isolates against B and S isolates are primarily related to the fructose and mannose metabolism, vitamin B6 metabolism, purine metabolism, valine, leucine, and isoleucine biosynthesis, phenylalanine metabolism, galactose metabolism, cysteine and methionine metabolism and aminoacyl-tRNA biosynthesis.

**Table 1:** The metabolic pathways analyzed with MetaboAnalyst 5.0 among up-regulated metabolites.

Pathway	R v S			R v B		
	Match	*p*-value	Impact	Match	*p*-value	Impact
**Fructose and mannose metabolism**	2/33	0.03	0.0168	3/33	0.00	0.0169
**Vitamin B6 metabolism**	1/13	0.11	0.0463	1/3	0.12	0.0463
**Purine metabolism**	2/73	0.14	0.0408	2/73	0.14	0.0409
**Valine, leucine, and isoleucine biosynthesis**	1/22	0.18	0.0000	1/22	0.19	0.0000
**Glycerophospholipid metabolism**	1/22	0.18	0.0562	1/22	0.19	0.0562
**Valine, leucine, and isoleucine degradation**	1/23	0.19	0.0000	1/23	0.19	0.0000
**Phenylalanine metabolism**	1/33	0.26	0.0000	1/33	0.27	0.0000
**Galactose metabolism**	1/39	0.31	0.0000	2/39	0.05	0.0000
**Cysteine and methionine metabolism**	1/40	0.31	0.0000	1/40	0.32	0.0000
**Aminoacyl-tRNA biosynthesis**	1/45	0.35	0.0000	1/45	0.35	0.0000

### Antibiotic resistance associated metabolites

Unique and significant metabolites that may be linked to antibiotic resistance, virulence, pathogenicity, and growth were significantly expressed in R isolates as compared to S isolates and B isolates (Table 2). These include L-Leucine, Pyridoxine (Vitamin B6), Indoleacrylic acid, D-Mannitol, Sphingolipids (Sphinganine, C16 Sphinganine, and C17, Sphinganine,), Dulcitol, and D-Proline. Nonetheless, 11,12-dihydroxy arachidic acid was common in B and R isolates (downregulated) which may be associated with the development of clarithromycin resistance in *H*. *pylori*. Interestingly, L-leucine (KEGG C00123) was involved in several metabolic pathways in the R isolates which includes valine, leucine, and isoleucine biosynthesis (map00290) and degradation (map00280), and aminoacyl-tRNA biosynthesis pathways (map00980). Furthermore, Pyridoxine (KEGG C00314) was present in the vitamin B6 metabolism pathway (map00750), and D-Mannitol (KEGG C00392) was present in the fructose and mannose metabolism pathway (map00051) (Fig 5). Additionally, few metabolites were downregulated in the R isolates and were upregulated in S and B isolates shown to have a role in diminishing the survival of bacteria [31,32]. Vitamin D metabolites [(25R)-1?,25,26-trihydroxy-22-oxavitamin D3, 23,24-didehydro-25-hydroxyvitamin D3, and 26,26,26,27,27,27-hexafluoro-1?a-hydroxyvitamin D3] were found to be downregulated in R isolates against S and B isolates (Table 2).

**Table 2:** The regulation of metabolites that correlate with bacterial survival and may constitute a potential antibiotic mechanism.

Metabolites	Comparisons								
	R v S			R v B			B v S		
	FC^1^	Reg^2^	*p*-value	FC	Reg	*p*-value	FC	Reg	*p*-value
**L-Leucine**	304.222	Up	0.000	3499.299	Up	0.035	-11.502	Down	0.049
**Pyridoxine (Vitamin B6)**	148.856	Up	0.190	1702.584	Up	0.023	-11.438	Down	0.018
**Indoleacrylic acid**	496.092	Up	0.192	74701.740	Up	0.010	-150.580	Down	0.012
**D-Mannitol**	26.524	Up	0.005	7687.407	Up	0.003	-289.826	Down	0.000
**Sphinganine**	179222.840	Up	0.423	179222.840	Up	0.022	-1.000	NSD^3^	0.176
**C16 Sphinganine**	448646.880	Up	0.423	448646.880	Up	0.016	-1.000	NSD	#DIV/0!
**C17 Sphinganine**	1259.546	Up	0.423	1259.546	Up	0.000	-1.000	NSD	#DIV/0!
**Dulcitol**	9.404	Up	0.001	704.470	Up	0.063	-74.913	Down	0.083
**D-Proline**	16.095	Up	0.180	5424.762	Up	0.016	-337.036	Down	0.000
**(25R)-1?,25,26-trihydroxy-22-oxavitamin D3**	-268.468	Down	0.258	-4808.623	Down	0.000	17.911	Up	0.000
**23,24-didehydro-25-hydroxyvitamin D3**	-29.546	Down	#DIV/0!	-23845.832	Down	0.000	807.067	Up	0.000
**26,26,26,27,27,27-hexafluoro-1?a-hydroxyvitamin D3**	-97.136	Down	#DIV/0!	-6511.752	Down	0.001	67.037	Up	0.000

^1^FC: Fold Change.

^2^Reg: Regulation.

^3^NSD: Non-Statistically Different.

## Discussion

Using the metabolomics approach, it is possible to identify chemicals or molecular targets that encourage antibiotic resistance and can be used in designing drugs against clinical isolates that are the focus of antibiotic treatment [9,33]. Untargeted metabolomics analyses, which enables the high-throughput characterization of a large scale of small molecule metabolites, is a useful discovery tool can provide novel and unbiased insights into the variations between drug-resistant and drug-sensitive strains [34]. Results from untargeted metabolomics can be very important in providing the basis for targeted analysis of specific metabolites or metabolic flux analysis of specific metabolic pathways [35].

In this study, a comparative global metabolome profiles of B and R isolates with parental the S isolates were investigated. The finding showed that more metabolites were expressed in B and R group compared to their parental S group. Nonetheless, B and S group expressed the most similar metabolites. Therefore, it could be suggested that R isolates may have produced more metabolites to enhance antibiotics resistance. This is in line with other finding that show the number of metabolites found in resistant mutants is threefold greater than the number of metabolites identified wild type [36]. This has also been demonstrated in *E*. *coli*, *Pseudomonas aeruginosa*, and *S*. *aureus*, which showed that drug resistant bacteria produce more metabolites when compared to drug sensitive strains [37–39]. It is crucial to note that the metabolic burden on a resistant pathogen relies highly on the adaptations to bacterial metabolism [8]. Moreover, bacteria producing more diverse metabolites can influence the antibiotic susceptibility of bacteria [40].

The involvement of bacterial metabolism in antibiotic resistance mechanisms and clinically relevant bacterial infections have been revealed by the cutting-edge metabolomics approaches. Amino acid metabolism, virulence, stress response, and cell-to-cell contacts in biofilms are examples of key cellular changes linked with antibiotic resistance in which metabolism plays a crucial role [9,17,41]. Interestingly, L-leucine, Pyridoxine (Vitamin B6), D-Mannitol, Sphingolipids, Indoleacrylic acid, Dulcitol, D-Proline were found to be significantly up-regulated in R isolates compared to S and B isolates. These metabolites may be associated with the development of antibiotic resistance in *H*. *pylori*.

In terms of essential nutrients for bacterial physiology, branched-chain amino acids such as Leucine (Leu) play a variety of roles, from promoting protein synthesis to signaling and support not only proliferation during infection, but also the evasion of host defenses [42–44]. It is also reported that Leu is essential for survival and bacterial growth [45]. Chemotactic responses of *H*. *pylori* to Leu were enhanced absolute requirement for growth [46]. Moreover, the biosynthesis of vitamin B6 has been shown to boost virulence in *H*. *pylori*, *Mycobacterium tuberculosis*, and *Streptococcus pneumoniae* [47]. Grubman et al. [48] demonstrated that mutant *H*. *pylori* deficient an enzyme PdxA, vital in vitamin B6 production were incapable to generate motility appendages and to establish chronic colonization in mice. This observation states that over-expression of vitamin B6 enhances virulence in bacteria which was also observed in the R isolates of this study.

D-mannitol is said to be a crucial component of the suitable solute system in osmoprotection in bacteria such as *S*. *aureus* [49]. In reaction to osmotic stress, mannitol-1-phosphate dehydrogenase (M1DPH) alters fructose-6-phosphate (F6P) to mannitol through the mannitol metabolism cycle [50]. M1DPH pathway was suggested as a target by Nguyen et al. [51] to establish an alternative treatment for Methicillin-resistant *S*. *aureus* (MRSA) infections. Furthermore, sphingolipids are found in the host cell membrane and act as building elements and signaling molecules which were reported to play an important role in the adhesion and invasion into the host cell for pathogenic *Neisseria* [52,53]. Although *H*. *pylori* has not been known to synthesize sphingolipid, other gut bacteria, such as *Bacteroides* and *Prevotella* spp., are known to produce sphingolipid to confer bacterial fitness in the gut [54,55]. Several studies have highlighted the role of sphingolipids as signaling molecules in the growth and pathogenicity of microbial pathogens including bacteria [56–59]. *H*. *pylori* have developed different strategies to manipulate and use host sphingolipids to promote their pathogenicity. It was reported that *H*. *pylori* uses glycosphingolipids as a receptor for adhesion and uptake [60]. This study showed that in response to clarithromycin stress, *H*. *pylori* generated sphinganine, therefore, its role in *H*. *pylori* needs to be further explored.

The functional role of indoleacrylic acid and dulcitol in *H*. *pylori* has not yet been revealed. However, its role in drug resistance and virulence in other Gram-negative bacteria has previously been reported. Indoleacrylic acid is a derivative of indole, an intercellular signaling chemical, which were reported be vital in the regulation of xenobiotic exporter genes, *mdtEF* and *acrD*, which increases drug resistance in *E*. *coli* [61]. Additionally, Nikaido et al. [62] proposed that indole increases drug resistance in *Salmonella enterica* by activating the efflux pump system via the transcriptional regulator. Additionally, the dulcitol fermentation is considered as a virulence factor [63]. A link was discovered between dulcitol fermentation and *E*. *coli* strain pathogenicity [64]. In mice, it was discovered that dulcitol-fermenting uroisolates were more nephropathogenic (but not deadly) than nondulcitol fermenting strains [65]. As a result, we postulated that both indoleacrylic acid and dulcitol might be involved or play a comparable role in drug resistance or virulence in *H*. *pylori*.

In addition, *H*. *pylori* uses proline as a carbon and energy source, and it flourishes in proline-rich microenvironments [66,67]. Studies has shown proline is a key role in *H*. *pylori* infection as stomach colonization requires proline utilization P (*puP*) [68,69]. It has also been shown that proline utilization A (PutA) proline metabolism increases oxidative stress tolerance in *E*. *coli*, indicating that proline metabolism needs to be further examined in the physiology and pathophysiology of *H*. *pylori* [70].

Therefore, the up-regulation of these metabolites (L-leucine, Pyridoxine (Vitamin B6), D-Mannitol, Sphingolipids, Indoleacrylic acid, Dulcitol, and D-Proline), that are linked to the development of antibiotic resistance, in R comparing to B and S may suggest a role or an association of those metabolites in enhancing the clarithromycin resistance to facilitate the *H*. *pylori* persistence [71]. Future research could explore more about the exact role of these metabolites in resistance mechanisms and its involvement in the microbial pathogenesis and virulence, that may offer a novel class of antimicrobial therapies to combat the ever-growing problem of antibiotic resistance [72]. Nevertheless, the downregulation of specific metabolites may assist bacteria in dealing with antibiotic stress [73]. Several vitamin D metabolites were shown to be downregulated in R isolates compared to S and B isolates, which have been shown to be significant in reducing bacterial overgrowth in the gut [31,32]. Therefore, it might be suggested that reduction of vitamin D production may enhance survivability of R isolates.

*H*. *pylori* infection plays a crucial role in the development of gastric cancer [4]. *H*. *pylori* can cause stomach epithelial cells to become inflamed, which can lead to the development of gastric cancer [71]. *H*. *pylori* can also directly affect epithelial cells, which can result in protein modification and DNA mutation [74]. However, metabolites produced by *H*. *pylori* have yet to be studied for their correlation with cancer development. The metabolites that were expressed in the R isolates were proven to play a role in cancer development and growth. For example, Leucine was shown to be a substrate for the development of cancer because its metabolism can affect a variety of biological functions, including protein synthesis and epigenetic control [75]. Additionally, sphingolipids, particularly sphingosine 1-phosphate (S1P), have become bio-effector molecules that promote angiogenesis, cancer cell development, and transformation [76]. Therefore, it is crucial to further study these metabolites and their correlation with cancer development, which will lead to the development of novel therapeutic strategies against human cancers [77].

## Conclusions

The study suggested that *H*. *pylori* may have undergone metabolomic reprogramming in the development of antibiotic resistance as B and R isolates produced more metabolites than S isolates. Moreover, we also suggest that several metabolites which include L-leucine, Pyridoxine (Vitamin B6), D-Mannitol, Sphingolipids (Sphinganine, C16 Sphinganine, and C17 Sphinganine), Indoleacrylic acid, Dulcitol, and D-Proline may have an association with the development of antibiotic resistant in *H*. *pylori*. The findings of this study could lead to a new strategy to discover new therapy to combat antibiotic resistance and could prevent antibiotic resistance development in *H*. *pylori*.

## Supporting information

S1 Fig Fold change of B and R compared to the parental S isolate. (TIF)

S2 Fig Pathway analysis of R isolates against S isolates. A) The up-regulated metabolites which were mapped to the metabolic pathways in the KEGG database. B) The down-regulated metabolites which were mapped to the metabolic pathways in the KEGG database.
(TIF)

S3 Fig Pathway analysis of B isolates against S isolates. A) The up-regulated metabolites which were mapped to the metabolic pathways in the KEGG database. B) The down-regulated metabolites which were mapped to the metabolic pathways in the KEGG database.
(TIF)

S4 Fig Pathway analysis of R isolates against B isolates. A) The up-regulated metabolites which were mapped to the metabolic pathways in the KEGG database. B) The down-regulated metabolites which were mapped to the metabolic pathways in the KEGG database.
(TIF)

S1 Table The regulations of the compounds expressed by S, B, and R *Helicobacter pylori* isolates. (XLSX)
