# Supplementary figures and images for: Exposure of Helicobacter pylori to clarithromycin in vitro resulting in the development of resistance and triggers metabolic reprogramming associated with virulence and pathogenicity
Source: PLoS One. 2024 Mar 6;19(3):e0298434. doi: 10.1371/journal.pone.0298434 (PMC10917248; doi:10.1371/journal.pone.0298434)

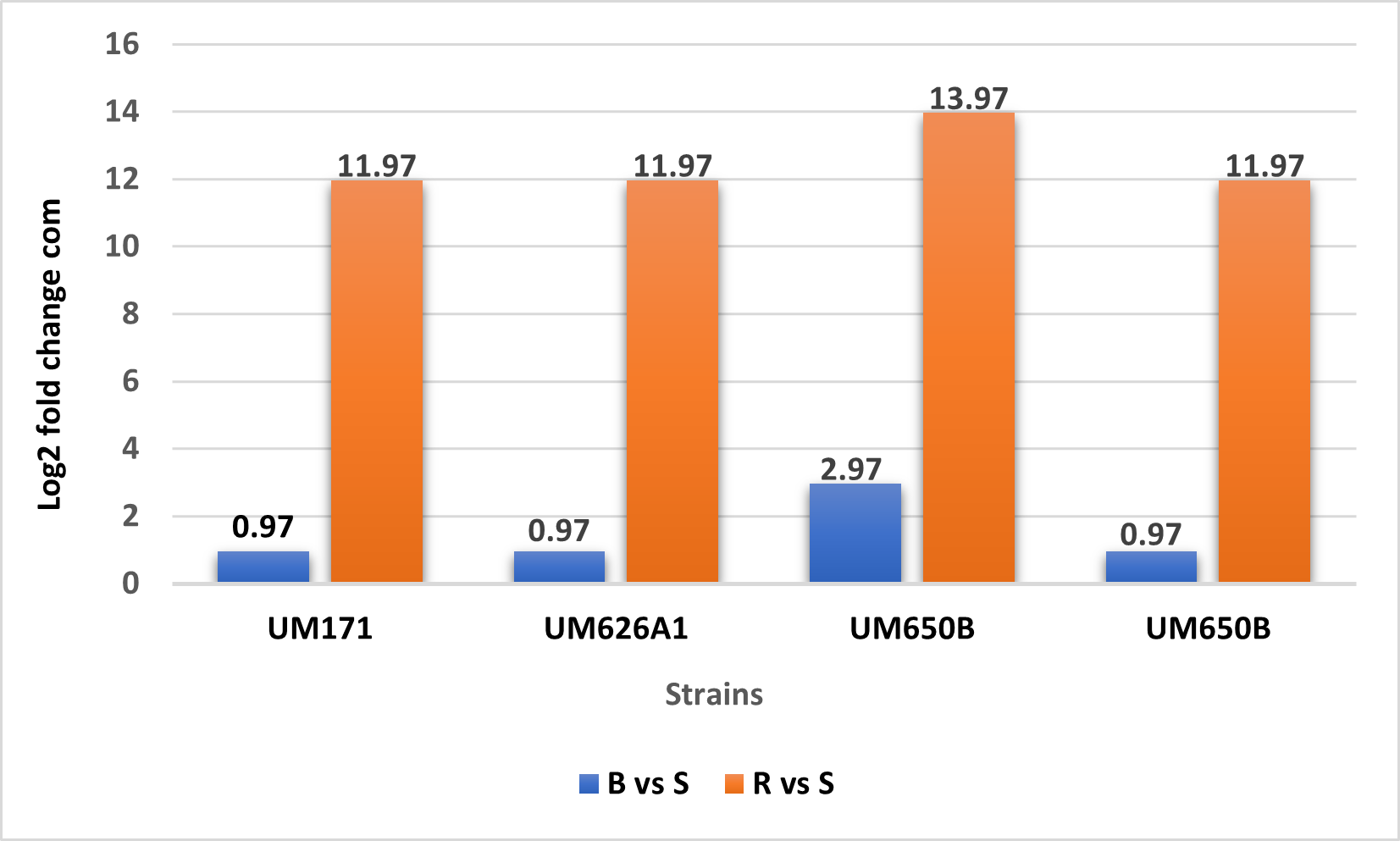

Supplement: S1 Fig — (TIF) [file pone.0298434.s001.tif]

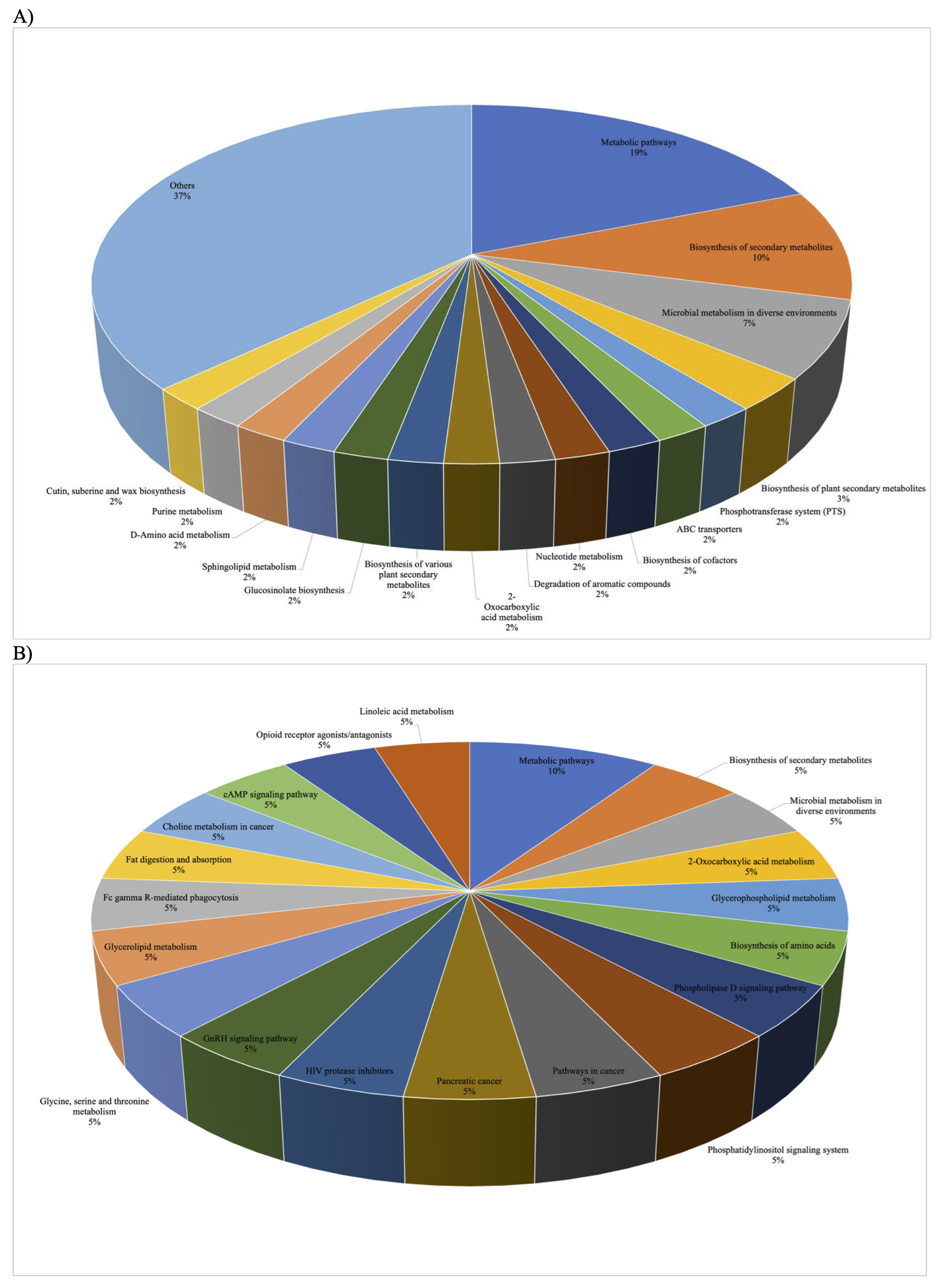

Supplement: S2 Fig — A) The up-regulated metabolites which were mapped to the metabolic pathways in the KEGG database. B) The down-regulated metabolites which were mapped to the metabolic pathways in the KEGG database. (TIF) [file pone.0298434.s002.tif]

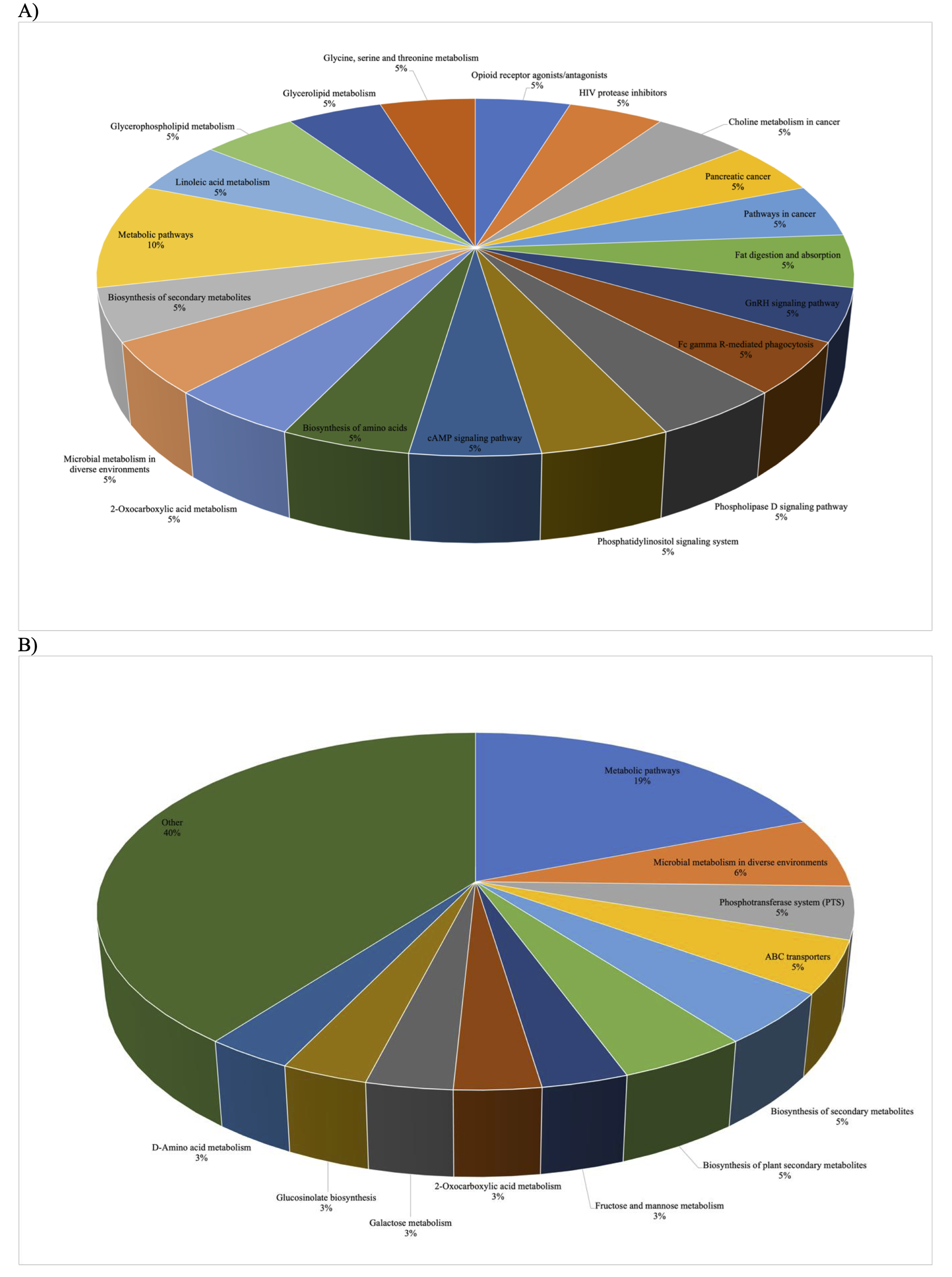

Supplement: S3 Fig — A) The up-regulated metabolites which were mapped to the metabolic pathways in the KEGG database. B) The down-regulated metabolites which were mapped to the metabolic pathways in the KEGG database. (TIF) [file pone.0298434.s003.tif]

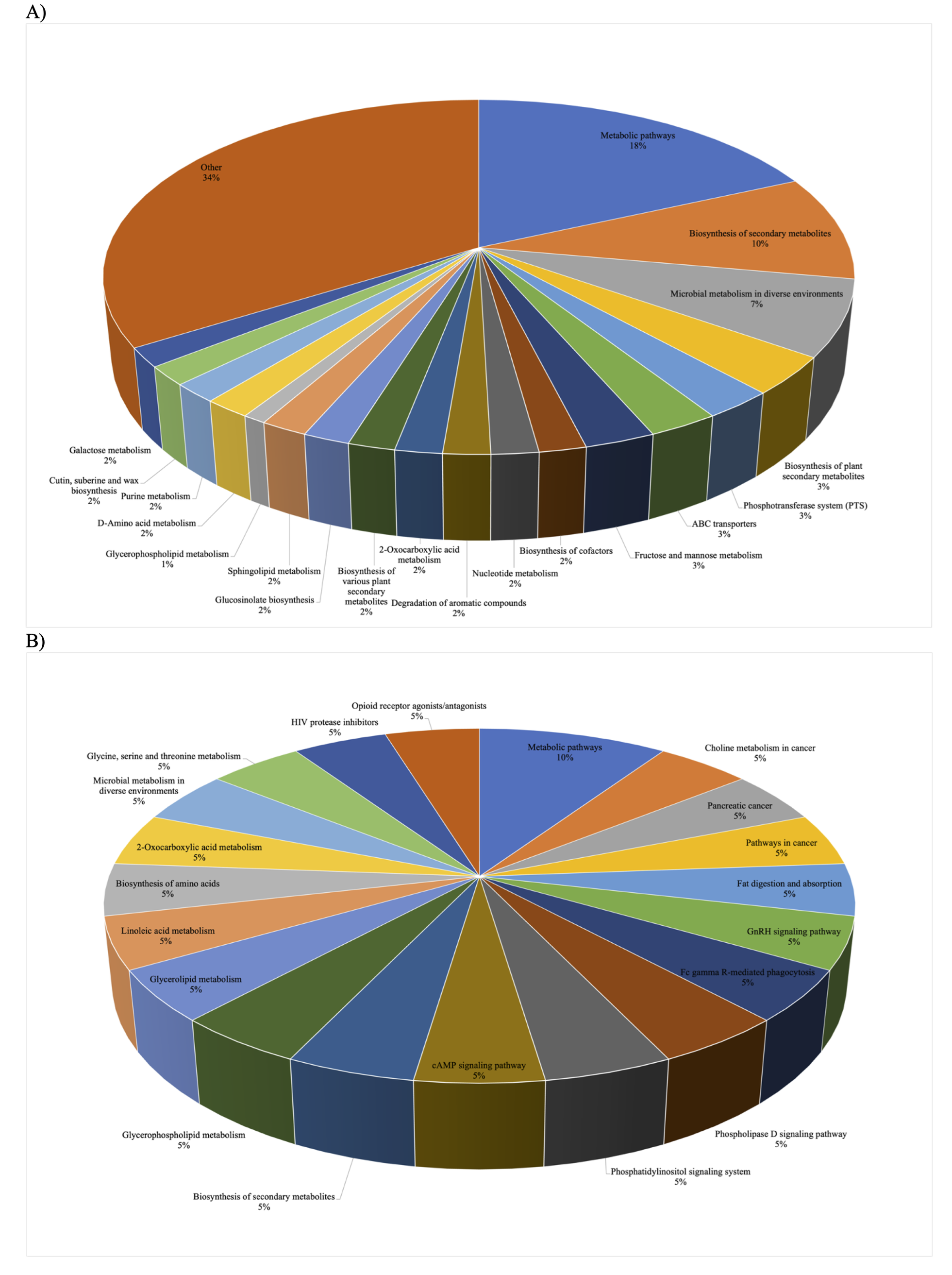

Supplement: S4 Fig — A) The up-regulated metabolites which were mapped to the metabolic pathways in the KEGG database. B) The down-regulated metabolites which were mapped to the metabolic pathways in the KEGG database. (TIF) [file pone.0298434.s004.tif]
